# Heritability Estimates of Traits Assessed in Field Performance Tests of Polish Warmblood Mares

**DOI:** 10.3390/genes17020148

**Published:** 2026-01-28

**Authors:** Dorota Lewczuk, Alicja Borowska, Małgorzata Maśko, Emilia Bagnicka

**Affiliations:** 1 Institute of Genetics and Animal Biotechnology PAS Jastrzębiec, Postępu 36a, 05-552 Magdalenka, Poland; 2 Department of Genetics and Animal Breeding, Poznań University of Life Sciences, Wojska Polskiego 28, 60-637 Poznan, Poland; alicja.borowska@up.poznan.pl; 3 Department of Animal Breeding, Institute of Animal Science, Warsaw University of Life Sciences SGGW, Nowoursynowska 166, 02-787 Warszawa, Poland; malgorzata_masko@sggw.edu.pl

**Keywords:** horse, breeding value, performance traits, jumping, riding, correlations

## Abstract

**Background/objectives**: Knowledge of the genetic background of evaluated traits has been the basis for genetic progress in every horse-breeding population and is essential for precise breeding and up-to-date decision-making. The study aimed to estimate the heritability coefficients for field performance traits in mares. **Methods**: The research was based on 1408 evaluations of mares conducted during the years 2002–2021 in 51 training centers in Poland. The preliminary analyses of the effects, significant for the investigated traits, were obtained using analysis of variance, and these additional data are also presented (SAS program, GLM and Mixed procedures). The final statistical model for the AI-REML procedure of the DMU program included a fixed effect of the training center-season-year of evaluation, a random effect of the animal, and a regression on age in years. **Results**: The heritability coefficients of the performance traits were moderate to high (0.32–0.60) with the SE within the range of 0.06–0.08. The highest heritability was estimated for the free jumping, trot, and overall results. The lowest heritability was achieved for the rideability. **Conclusions**: The level of heritability estimations allowed for the population progress in the evaluated traits.

## 1. Introduction

Knowledge of the genetic background of evaluated traits is the basis for genetic progress in every horse-breeding population [1] and is essential for precise breeding and up-to-date decision-making. Thus, the continued evaluation process is a key component of the breeding strategy. The genetic background of the traits of the breeders’ interest has been monitored in sport-horse breeding since the early 80s of the last century [2,3,4,5,6,7,8]. Then, it was developed for many breeds and kinds of performances, mainly in the sport horse [9], as well as for racing or trotter populations [10,11]. Even if not all horse breeding associations predict breeding value [12], it remains significant information on the proportion of the genetic variance in the general variability of the horse’s estimated traits. Among 26 national associations belonging to the World Breeding Federation for Sport Horses that answered the questionnaire, 18 associations have breeding value predictions for the traits of breeding goals. In Poland, official breeding value prediction started in the late 1990s of the 20th century [13], and the mare performance tests, being one of the first breeding steps, were often the subject of investigation [14,15,16]. Most papers discuss the breed effect influencing the results, which is not the subject of the current investigation, as warmblood horses in Poland are treated nowadays collectively as sport-registered horses. The heritability of the traits was investigated by Borowska [17] and Maśko [18]. In the first publication, the station performance tests were analyzed for mares, so the comparability of data is limited as the stationary test data and stallion test data are characterized by the higher heritability coefficients [17]. The second publication [18] covers field mares’ performance tests from 2002 until 2012, hence the update of the genetic background for the traits under selection is necessary to address changes in the pedigrees, as more foreign horses were imported to Poland. In Polish breeding value, however, not all sources of information are used, as the sport data is not included, except for some research purposes [19,20,21,22], mostly because of the lack of an integrated official result system. Breeding value prediction in Poland is not easy to implement because of the number of changes in the testing procedures over the years that do not allow for a continuous process of breeding value prediction. Based on the possible sources of information, nowadays breeding value prediction is mainly conducted by using the field performance tests of mares. The amount of data (above 1000 evaluated mares) allowed for the calculation of the heritability and breeding value prediction for traits usually evaluated on young horses’ performance tests. These traits are of special importance as they are closely connected with the main aim of the breeders—sport performance [23,24,25,26]—especially if the same kind of horse ability, dressage vs. jumping [27], is considered. This study aimed to estimate the variance components to achieve the heritability coefficients of the traits of mare field performance as one of the basic selection procedures, as well as present correlations between phenotypic and genetic evaluations. The results obtained in our study would allow for the comparison of the warmblood performance genetic background across other European countries and give a wider overall view of this characteristic at the European level.

## 2. Materials and Methods

### 2.1. Horses and Pedigree Characterization

The research is based on 1408 evaluations of mares (with some repetitions) conducted during the years 2002–2021 in 51 training centers in Poland. Training centers for the conduction of the one-day tests are selected by the Polish Horse Breeders Association based on their environmental suitability (riding halls, number of stables, paddock’s quality, riding areas, and personnel availability). The number of mares evaluated each month is presented in Table 1.

The mares are preselected based on conformation and are comparable in conformation traits. The mares must be at least 157 cm at the withers, with a minimum of 6 conformation points in the Polish linear profiling system, including also some subjective evaluation (like in the Dutch KWPN horse breeding linear sheet). Thus, “type” and “exterior” traits must be at least 6 on a scale of 1–10 (1-very bad; 10-excellent). The age of the mares must be at least three years. At present, the performance test can be repeated without an age limit, but younger horses attend the tests more frequently. The mean age was 4.41 (SD = 1.9; from 3 to 17). The mares could repeat the test; however, the number of mares that repeated the test was only 25 mares in the final file of 1408. Mares evaluated in the presented study were bred by 640 stallions. There are 12 popular sires with more than 10 offspring participating in the performance test, with a maximum of 27 mares. Among 640 sires of mares, 84 stallions had between 4 and 10 daughters; 160 sires had between 1 and 3; and 400 sires had only 1 representative. During evaluation, mares were not presented in pedigree groups, but the judges had pedigree information about each horse.

The pedigree of investigated horses was evaluated using the CFC 1.0 program (2006; http://agrews.agr.niigatau.ac.jp/~iwsk/cfc.html, accessed on 4 December 2025). The pedigree descriptions of the whole investigated population were achieved for 1394 horses. Within this population, 714 horses were inbred. The mean inbreeding coefficient was 0.545% (range from 0.002% to 17.85%) for all horses and 1.64% for inbreds. The mean coefficient of co-ancestry was 0.376, and 4076 horses were found as founders. The coefficient of co-ancestry was above 0.5 for 19 founders, above 0.2 for 67 founders, and above 0.1 for 106 founders. The average discrete generation equivalent in the analyzed population was 5.22. The database consisted of 327,730 records, and an extracted pedigree was obtained for 15,454 horses. The data for the period of the last 20 years is presented in Tables S1 and S2 to characterize the connections between the evaluated population.

### 2.2. Test Procedures

The mares are tested for one day, mostly in autumn. First, they are tested in free jumping in the jumping line with the oxer/double barre as the last obstacle and two small obstacles to allow the horse to correct distances between strides of canter. After the jumping test, horses are judged for quality of gait under the rider in walk, trot, and canter in the riding hall. The gait evaluations consist of the following: working trot with the evaluation of the possibility of lengthening the strides on the diagonal of the riding hall; working canter around the riding hall with a starting point from the corner of the riding hall, with the simple change of leading leg in canter, done in trot; extended walk around the hall and free walk allowing the horse to stretch on a long rein—“chewing the reins out of the hand” exercise. At the end of the test, a test rider (a person unfamiliar with the horse, selected from a list of people certified by the Polish Horse Breeders Association to test young horses) rides them and gives notes on their rideability. The following five evaluations are given:

1. Free jumping—style, reflex, jumping ability, courage, easy to jump;
2. Work in walk—energy, length of the stride, regularity of strides;
3. Work in trot—energy, length of the stride, regularity of strides;
4. Work in canter—energy, regularity of strides, lightness of the front, engagement of the croup.
5. Rider’s evaluation (rideability)—evaluation of cooperation with the test rider.

The overall result is the sum of all traits evaluated with an interval of 0.5 points. Because during the investigation period the scale of the overall sum has changed, we have used the percentage of the possible value to be achieved as the final result for the calculations. The scale used for every trait evaluation ranged from 1 to 10 points (1—very bad; 10—excellent). The final result scale was once expressed as the sum of all traits, and once as the mean of all traits. Horses can be withdrawn from the test in the case of lameness. The judges’ committee consisted of persons schooled by the Polish Horse Breeders Association. The test riders were selected by the test organizers from a list of experts schooled and allowed to judge the horses in these tests.

The basic statistics of the presented traits are given in Table 2. The coefficients of variation, as shown in Table 2, do not exceed 15%, ranging from 9.56% to 14.8%. Such characteristics are obvious in sport horse breeding, and the normal distribution is assumed [23]. All effects that could be identified were considered in the creation of the statistical model.

### 2.3. Preliminary Statistical Analyses of Phenotypes

The statistical model for data analysis was based on analysis of variances, first performed using the GLM procedure of the SAS package (SAS/STAT, 2002–2012, version 9.4), then using the MIXED procedure with the additional sire effect from the same statistical package. The data were analyzed by different models and procedures to ensure the stability and properties of the statistics used. If the results are comparable and/or the differences understandable, the correctness of the model seems adequate. The analysis of variance included the fixed effects of the training center, year, and season of evaluation. The season was created based on the months of the test, because of the unequal distribution of mares’ testing in the single months (Table 1). The age of horses (in years) was used as the linear regression as fitted in the preliminary analyses. The Tukey post hoc tests were used to evaluate differences between levels of effects. The following model was used for preliminary data investigations:

y_ijkl_ = µ + Y_i_ + TC_j_ + S_k_ + (ß × age)_ijkl_ + e_ijkl_ (1)

where

y_ijkl_—trait evaluation,
µ—population mean of the trait,
Y_i_—fixed effect of year (i = 2002, …, 2021),
TC_j_—fixed effect of the training center (j = 1, …, 51),
S_k_—fixed effect of season (k = 1, …, 4),
(ß × age)_ijkl_ —linear regression on the age in years,
e_ijkl_—random residual effect.

The second statistical model taken into analysis was used in the MIXED procedure of the SAS program, and the random sire effect was added but without pedigree connections. There were 640 sires of 1408 mares investigated in the study. The stallion influence was presented in results using the repeatability of the trait. This parameter was expressed as the ratio of the sire effect to all random effects. The Tukey post hoc tests were used to evaluate differences between levels of effects. The model for the Mixed procedure was as follows:

y_ijklm_ = µ + s_i_ + Y_j_ + TC_k_ + S_l_ + (ß × age)_ijklm_ + e_ijklm_ (2)

where

y_ijklm_—trait evaluation,
µ—mean of the trait,
s_i_—random effect of the sire (i = 1, …, 640),
Y_j_—fixed effect of year (j = 2002, …, 2021),
TC_k_—fixed effect of the training center (k = 1, …, 51),
S_l_—fixed effect of season (l = 1, …, 4),
(ß × age)_ijklm_—linear regression on the age in years,
e_ijklm_—random residual effect.

### 2.4. Statistics for Genetic Analyses

After preliminary analyses, we have selected statistically significant effects for the final model. However, because the results of mares were strictly connected with the training center and year of the test, the combined effect of the training center-season-year was used. The effect of the herd-year-season of the test is usually used in the models for breeding value predictions in many farm animals. In this model, the age of horses was used as the linear regression in years because it was significant for most traits analyzed earlier. The animal model with the AI-REML method was used in the DMU package of Madsen and Jensen [28], and the single-trait models were used. The final model for the heritability coefficient estimation is presented below, and the pedigree information (a relationship matrix) was included:

y_ijk_ = µ + a_i_ + (TC × S × Y)_j_ + (ß × age)_ijk_ + e_ijk_ (3)

where

y_ijk_—trait phenotypic value;
µ—mean of the trait;
a_i_—random direct additive effect of the i-th animal a ~N (0, Aσ_a_^2^), where mean = 0, A is an additive relationship matrix, and δ_a_^2^ is the additive genetic variance (i = 1, …, 1408 mares with pedigree);
(TC × S × Y)_j_—combined fixed effect of the training center × season × year (j = 1, …, 4008);
(ß × age)_ijk_—linear regression on the age in years;
e_ijk_—residual random error associated with each observation ~N(0, Iσ_e_^2^), where mean = 0, I is identity matrix, σ_e_^2^ random error variance.

We assumed the normality of the distribution for random effects; thus, a_i_ is the random i-th animal effect ~N(0, Aσ_a_^2^), while e_ijk_ in all models is the random ~N(0, Iσ_e_^2^) residual effect. Although some animals had repeated observations, we did not use the permanent environmental effect in the model, because this only concerned 25 mares (1.77% of animals), and we considered that it did not bias the results.

Additionally, the correlations between phenotypic evaluations, as well as the correlations between genetic value estimations, were conducted. The Pearson correlations (PROC CORR, SAS/STAT, 2002–2012, version 9.4) were used in both cases. The breeding value prediction was achieved for the investigated horses using the above final model using AI BLUP in the DMU package [28].

## 3. Results

The investigated population was genetically connected. The inbreeding coefficient was found to be reasonable, especially for certain years, such as 2014 (1.2%). Other high values (>0.7) are noted in different years—the first in 1999 and the last in 2011, 2014, and 2018. The coefficient of co-ancestry, as expected, has been growing with the years, especially in the last three years of the investigated horses’ birth years.

Investigated traits variances were characterized by the model used at a rather moderate level, as the coefficients of determination were between 11 and 25% and the highest values, 22–26%, were obtained for the overall results, expressed as a percentage of the maximum value, and the rider evaluation (Table 3). The effects of the investigated factors are presented in Table 3 for the GLM procedure and Table 4 for the Mixed procedure.

Both analyses showed that for the free jumping trait, the training center of the test has a statistically significant effect (*p* < 0.009), as does the age of the mares (*p* ≤ 0.01). The year of the test was statistically significant (*p* = 0.007) for the GLM procedure (without sire effect) and was almost the same, *p* = 0.008, for the Mixed procedure with the sire effect.

The analyses performed for the trait “walk” showed that the effects of the training center, year of test, and season were significant (*p* < 0.0001) in both cases. The significance of the regression on age was not confirmed in both analyses. Almost the same results were achieved for the “trot” trait. The effect of the training center-season-year was significant for *p*-value at least 0.008 for both analyses. The age did not influence the trot movement. The last movement trait evaluated on the performance test, “canter,” was significantly dependent at the *p* < 0.0001 level on the training center and year of the test effects, and lower for *p* < 0.02 for season and age effects in the case of GLM and Mixed analyses. The evaluation of the test rider was affected by the training center and year of the test (*p* ≤ 0.009) and age (*p* ≤ 0.05).

The overall evaluation of mares—the total results expressed as the percentage of the maximum possible points—was influenced by all investigated effects (*p*-value lower than 0.0089). The age was statistically significant at a high level of *p* < 0.0001 for the GLM procedure and for the Mixed procedure. Comparing both procedures and models, it seems that the introduction of the sire effect changed the meaning of the year effect, although not substantially. Because of the close connections between the quality of the sires and genetic progress within the following years, which is the usual breeding process, it seems natural and understandable. The repeatability of the trait evaluation calculated from the sire effect was medium or low, reaching from 0.25 to 0.32 for the overall results and free jumping traits from 0.18 to 0.24 for movement traits, with the lowest value for the riders’ evaluations (0.12) (Table 4).

The heritability coefficients calculated based on the presented model were from medium to high values (Table 5). The higher level of h^2^ = 0.60 (SE 0.08) was achieved for the trait free jumping and the overall result; the lowest value was achieved for the rider evaluation h^2^ = 0.32 (SE 0.08). The heritability coefficient for movement traits was achieved at a high level of 0.50. The regression on age was characterized by the negative value of the regression coefficient for all traits.

The calculated correlations were presented in Table 6. They are of high and moderate levels, with the highest value for both phenotypic and genetic correlations between results in % and almost all other traits (0.70–0.86). The lowest values were noted between free jumping and all other traits (0.33–0.49).

## 4. Discussion

Pedigree analyses showed that data for mares evaluated on the field tests are comparable with the coefficients obtained for sport horses attending show jumping competitions [22]. These pedigree data should be monitored in every sport horse population, because of the limited gene pool selection conducted by breeders.

The stability of the effects investigated in both analyses and procedures seems comparable, as most effects are of the same significance in both models. The observed small changes between GLM and Mixed procedures seem understandable, as the quality of sires is connected to the year, and not all sires are observed in all years. The heritability coefficients estimated using an animal model with pedigree relationships followed the trend observed for the repeatability coefficients estimated using the sire model. The analysis of factors influencing the traits studied gave comparable results with other European evaluations [13]. The training center in our result or competition (Belgium, Denmark) or group of comparison (Germany) is often used in the model for breeding value prediction as a significant factor. Even if it is not always the same definition of the group, it allows us to exclude the environmental effect from the genetic background. The same relates to the effect of the year being widely used in estimation, as the year of testing (Germany, France) or the year of the animal’s birth (Sweden). This effect is also indirectly considered in many estimations [13] as a “rolling base” and is often used as a reference population. The “rolling base” can relate to age—“horses between 4 and 18 years” (Ireland, Sweden), “horses between 7 and 18” (Belgium), “between 3 and 17” (Denmark), “between 11 and 15” (Germany), “born 5 years before evaluation” (France). The season of testing is seldom used in other countries, because in many countries the tests are organized in the same season. Probably the “competition effect” covers that part of the variance. In our case, the effect of the season of testing is significant, as evaluated earlier [29]. However, the season could be expected to be more significant, also for the traits of free jumping and rider evaluation. Most of the tests were conducted in autumn; there is a possibility that the horses are being prepared inside the riding halls for most traits. The period of test observation is also long (20 years) and covers economic changes in breeding structure, which allow the modification of some seasonal effects, which was not the case in Poland in the early years of 2000. The seasonality of the horse’s physiology and even the daily rhythm of horses are known. Because of the limited number of horses that can be included in the evaluation to allow for a more precise evaluation, the season, year, and the training center of the test were included in the statistical model as the joint class effect.

The age of horses is extremely important for the horse evaluation [30,31,32,33,34,35] and that is why it is included in most evaluations of breeding values in jumping evaluations [12]. In some countries, it relates to the last age when achievements were recorded (Ireland, Germany) by sports competition data. In most evaluations, it is used as the age class in connection with the sex effect (Denmark, Germany) or individually, as in our case (France, Belgium). The age of horses was estimated as significant in our study for half of the traits and finally for the overall result. Even if the age effect is not significant for all traits, in all models, we decided to leave this effect in the final model. The low significance of the age effect in some cases could be explained by corrections that judges or riders can make by evaluation, even unconsciously. They discern age and month of horse birth by evaluation; most judges are trainers and such corrections on age may come automatically as a part of their job. The differences between judging horses of different ages were stated [36].

It can be expected that, usually, with more years of age and more training, the evaluation should be better. It was not the case with our results, as the regression analyses were negative. It might be possible that breeders/owners presented their horses at a later age because of some earlier injuries or a lack of proper form. On the other hand, the training period, not known by field performance tests, is more significant than the age itself [37].

The stallion (sire) effect, expressed as repeatability, was mostly at a low to medium level. However, repeatability for the overall and free jumping was higher. The heritability coefficients, estimated based on greater pedigree information, were higher. Thus, breeding progress is possible. Some future problems may arise, as there was a tendency, mentioned earlier [22,38], to use popular stallions more intensively. In our research, we can distinguish several stallions that were used frequently for breeding. This, unfortunately, may cause a reduction in the genetic diversity of the population.

The data obtained for the breeding value prediction is in the range of European known values, as well as comparable in trends with earlier estimations. According to the European review [23], the highest value for the heritability coefficient of the field performance tests for mares was obtained for the jumping ability, as in our results. The European mean reached 0.37 [23]; however, the highest values were for the Netherlands, reaching up to 0.62. Almost the same value of the mean heritability coefficient (0.35) was observed for the trot and all other movement traits. In our results, the same heritability coefficients were about 0.5. A similar value of the heritability coefficient was observed in the Netherlands, and a little lower in Sweden. In most countries, the values for the heritability coefficient of the walk and canter were a little lower than in our research and reached 0.28–0.30 as a mean [23] and 0.35–0.39 were the highest values. The heritability coefficient of the rideability was, as a European mean (0.21) and as the individual countries’ estimations (0.003–0.30), the lowest value among all estimated. In our research, it also reached the lowest value of 0.32. The same tendency was observed in other studies [39,40,41,42]. The same trait heritability coefficient was estimated as one of the highest during the introduction of this rideability trait in the early 80s [3]. The lowering of the heritability coefficient through the years can relate to the problem of the trait’s definition stressed by von Borstel et al. [43]. However, the problem of trait definitions is not a new one [1,12,44]. In Poland, rideability trait was estimated earlier at a comparable level (0.34–0.38) as estimated by Borowska [17] and Maśko [18]. The heritability coefficient value estimated for the rider evaluation in our study corresponds with the data above. In the field performance test results obtained by Maśko [18] for the mares up to the 2012 testing year, the trot evaluation is on the same value as in our result (0.50); however, the heritability coefficients of the walk and canter evaluations are a little lower, being 0.4 and 0.3, respectively. Almost the same results as in our study were observed in the study of Borowska [17] on the station performance tests. The heritability of the free jumping, work in trot, and the final results were the highest heritable traits. According to some scaling, they could be considered even higher. The heritability of the performance traits is supposed to be overestimated because of the judges’ corrections based on their expectations from the horse catalog pedigree information [45]. All obtained and discussed results may be influenced by the preselection of horses based on their conformation; this problem concerns all populations because the conformation selection is the first step of selection in every warmblood breeding association. The number of animals is not huge; however, we hope that it gives the first preliminary view on the structure of the variance in the Polish warmblood population. The study will be conducted further.

The genetic correlations were higher than the phenotypic ones; in the case of overall results, the same was observed for the free jumping trait and the rider’s evaluation. The correlations between genetic values may not be the same as those calculated using a multi-trait model using phenotypic data [46]; however, the trends in differences between correlations can still be observed and compared. The correlations between breeding values for the jumping and movement traits are positive, which is not observed in more specialized populations [27,45], probably because of the level of performance effort. The requirements of movement and jumping needed and checked on the performance test are much lower than on dressage and show jumping competitions, as well as the specialization only being provided in Poland since 2017. Thus, the investigated population is rather an all-rounder sport horse.

The changes in heritability should be monitored continuously for breeding purposes. Some information on the trait heritability may not change spectacularly. Higher conformation heritability than performance, once it is known and stable [47], even new, more detailed genomic information appears [48,49]. Continuous monitoring of heritability coefficients for traits with the use of new, genomic methods [50,51] seems reasonable also on the European level, as the genetic connectedness between horses is meaningful [52]. Finding new traits describing the horse evaluation earlier [53,54] or more objectively could help in the horse breeding programs, as it was underlined for sport horse breeding [1,12,55,56] for many years and realized on different levels [55,56,57,58,59].

## 5. Conclusions

The age of the horse is a significant factor influencing the performance test results, at a different level, perhaps due to possible conscious or unconscious corrections of judges and riders. The season of the evaluation plays an important role in the horse evaluation; however, it is less regular than expected. The heritability coefficients of the performance traits are moderate or high. The highest heritability estimate was obtained for the free jumping, trot, and overall results. The lowest heritability was achieved for the rideability, and it may be connected to the subjective trait definition and evaluation. The heritability estimates are in the range of earlier calculations, so the population genetic progress still seems possible.

## Figures and Tables

**Table 1:** Number of mares tested in months and seasons.

Month	n	Season	n
December	60	winter	262
January	116	winter	262
February	86	winter	262
March	53	spring	203
April	62	spring	203
May	88	spring	203
June	230	summer	437
July	193	summer	437
August	14	summer	437
September	85	autumn	506
October	166	autumn	506
November	255	autumn	506
All	1408	all	1408

**Table 2:** Basic statistics of evaluated traits given in points.

Traits	n	Mean Points	Standard Deviation	Minimum	Maximum
Free jumping	1395	7.28	1.03	3	10
Walk	1395	6.89	0.81	3	10
Trot	1395	6.91	0.83	3	10
Canter	1395	6.93	0.82	3	10
Rider evaluation	1392	7.10	1.06	1	9
Result—% of max	1408	69.68	7.51	16.76 *	98

* Horses did not have to attend all traits if they were withdrawn from the tests.

**Table 3:** Effects influencing results of mare’s performance analyzed using GLM procedure.

Traits (1–10 Points)		Effects			
		Training Center (1–51)	Season *	Year (2002–2021)	Age in Years (3–17)
Free jumping R^2^ = 0.11 CV = 13.7%	LSM Min-max	5.79–8.36	7.07–7.47	6.84–8.68	-
	*p*-value	0.0051	0.1134	0.0077	0.0166
Walk R^2^ = 0.18 CV = 10.9%	LSM Min-max	6.07–8.24	6.63–7.11	6.49–8.69	-
	*p*-value	<0.0001	<0.0001	<0.0001	0.1155
Trot R^2^ = 0.19 CV = 11.05%	LSM Min-max	6.17–7.98	6.80–7.15	6.46–7.46	-
	*p*-value	<0.0001	0.0043	<0.0001	0.3759
Canter R^2^ = 0.20 CV = 10.9%	LSM Min-max	6.15–8.68	6.84–7.00	6.62–7.38	-
	*p*-value	<0.0001	0.0147	<0.0001	0.0203
Rider evaluation R^2^ = 0.21 CV = 14.8%	LSM Min-max	6.10–8.87	6.92–7.31	6.40–7.96	-
	*p*-value	<0.0001	0.0781	0.0003	0.0529
Results (% of max) R^2^ = 0.25 CV = 9.56%	LSM Min-max	49.68–78.47	66.66–70.14	63.23–70.50	-
	*p*-value	<0.0001	0.0016	<0.0001	<0.0001

* Season—winter: December–February; spring: March–May; summer: June–August; autumn: September–November. R^2^—coefficient of determination of the model used, CV—coefficient of variation for the trait.

**Table 4:** Factors influencing results of mare’s performance analyzed using MIXED procedure.

Traits (1–10 Points)		Effects				
		Training Center (1–51)	Season * (1–4)	Year (2002–2021)	Age in Years (3–17)	Sire (1–640)
Free jumping	LSM Min-max	5.94–8.31	7.15–7.43	6.80–7.75	-	R = 0.25
	*p*-value	0.0099	0.3606	0.0081	0.0044	
Walk	LSM Min-max	6.11–8.20	6.64–7.10	6.48–7.27	-	R = 0.17
	*p*-value	<0.0001	<0.0001	<0.0001	0.1636	
Trot	LSM Min-max	6.10–8.07	6.80–6.92	6.41–7.61	-	R = 0.24
	*p*-value	<0.0001	0.0082	<0.0001	0.4733	
Canter	LSM Min-max	6.13–8.61	6.81–7.09	6.60–7.37	-	R = 0.18
	*p*-value	<0.0001	0.0200	<0.0001	0.0209	
Rider evaluation	LSM Min-max	6.01–8.88	6.93–7.29	6.39–7.98	-	R = 0.12
	*p*-value	<0.0001	0.1522	0.0009	0.0495	
Results (% of max)	LSM, min-max	49.74–79.18	66.97–69.90	63.00–70.37	-	R = 0.32
	*p*-value	<0.0001	0.0089	<0.0001	<0.0001	

* Season—winter: December–February; spring: March–May; summer: June–August; autumn: September–November. LSM—least square means; *p*-value—significance level from the tests; R—repeatability.

**Table 5:** Heritability coefficients and results for the regression on age.

Trait	σ_a_^2^	σ_e_^2^	h^2^	SE	Regression on Age in Years	SE for Regression
Free jumping	0.59	0.39	0.60	0.08	−0.02912	0.016
Walk	0.26	0.29	0.47	0.08	−0.01714	0.012
Trot	0.29	0.28	0.50	0.08	−0.00600	0.012
Canter	0.26	0.29	0.47	0.07	−0.23144	0.012
Rider’s evaluation	0.34	0.73	0.32	0.08	−0.00360	0.017
Results (% of max)	24.78	16.54	0.60	0.08	−0.42258	0.010

σ_a_^2^—additive genetic variance; σ_e_^2^—random error variance; h^2^—heritability coefficient; SE—standard error.

**Table 6:** Correlations between phenotypical evaluation (above the diagonal) and genetic breeding value for traits (below the diagonal) between mare’s field performance traits (*p* < 0.0001).

Correlations	Result %	Free Jumping	Walk	Trot	Canter	Rider’s Evaluation
Results (% of max)	x	0.64	0.67	0.74	0.78	0.76
Free jumping	0.70	x	0.33	0.37	0.44	0.39
Walk	0.71	0.34	x	0.59	0.61	0.42
Trot	0.79	0.38	0.57	x	0.76	0.46
Canter	0.86	0.49	0.59	0.75	x	0.54
Rider evaluation	0.80	0.44	0.46	0.55	0.63	x

## Data Availability

The data used for calculation belongs to the Polish Horse Breeders Association.

## Supplementary Materials

The following supporting information can be downloaded at: https://www.mdpi.com/article/10.3390/genes17020148/s1, Table S1: The coefficients of inbreeding for the investigated population; Table S2: The number of founders and coefficients of co-ancestry for the investigated population.
