# Supplementary material for: Heritability Estimates of Traits Assessed in Field Performance Tests of Polish Warmblood Mares
Source: Genes (Basel). 2026 Jan 28;17(2):148. doi: 10.3390/genes17020148 (PMC12940987; doi:10.3390/genes17020148)
Supplement: Supplementary file 1 [file genes-17-00148-s001.zip › genes-4061931-supplementary.pdf]

genes

MDPI

SUPPLEMENTS

Table S1.

The coefficients of inbreeding for the investigated population

Year of birth	N	N inbreds	F%	F% inbreds	Maximum* F%	Minimum* F%
1999	50	13	0.733	2.819	14.792	0.049
2000	64	19	0.298	1.005	3.125	0.049
2001	72	25	0.613	1.766	18.750	0.012
2002	103	30	0.449	1.540	6.836	0.049
2003	83	36	0.281	0.647	5.249	0.003
2004	94	36	0.409	1.068	6.250	0.003
2005	71	33	0.256	0.551	3.516	0.006
2006	82	43	0.411	0.783	3.320	0.012
2007	62	35	0.449	0.795	6.860	0.006
2008	64	34	0.460	0.866	4.736	0.012
2009	78	42	0.531	0.987	4.980	0.002
2010	90	48	0.529	0.992	7.037	0.024
2011	68	47	0.902	1.306	14.356	0.012
2012	73	56	0.827	1.078	13.025	0.006
2013	69	48	0.685	0.985	4.462	0.014
2014	50	38	1.231	1.620	8.020	0.024
2015	52	37	0.686	0.964	12.708	0.003
2016	35	27	0.426	0.553	3.381	0.031
2017	20	16	0.842	1.053	3.781	0.024
2018	14	10	0.777	1.087	4.004	0.061

N – number of horses; N inbreds – number of inbreeded horses; F%- coefficient of inbreeding in %; F% inbreeds - coefficient of inbreeding in % for the inbreeded horses; *minimum and maximum values of F% are given for the whole population.

Table S2.
The number of founders and coefficients of co-ancestry for the investigated population

**Year of birth**	**N**	**N founders**	**f_e**	**f_{ge}**	**N_{enf}**	**f%**
1999	50	716	259	31	35	1.612
2000	64	909	308	46	54	1.084
2001	72	1060	355	49	57	1.021
2002	103	1179	337	58	70	0.865
2003	83	1182	337	51	61	0.972
2004	94	1166	282	47	57	1.054
2005	71	1234	362	47	54	1.066
2006	82	1288	316	46	53	1.097
2007	62	1208	325	34	38	1.478
2008	64	1203	313	39	45	1.271
2009	78	1313	306	43	51	1.152
2010	90	1378	318	45	53	1.110
2011	68	1211	263	37	43	1.341
2012	73	1445	254	38	45	1.306
2013	69	1266	316	38	44	1.303
2014	50	957	233	26	29	1.929
2015	52	1173	253	30	34	1.679
2016	35	922	272	24	26	2.088
2017	20	584	187	14	15	3.686
2018	14	461	138	10	11	5.085

N- number of horses; N founders -number of founders for the group of horses; f_e - effective number of founders; f_{ge} - effective number of non-founders; %; N_{enf} - non-founder equivalent; f% - coefficient of co-ancestry.
